# Neonatal Circulating Amino Acids and Lipid Metabolites Mediate the Association of Maternal Gestational Diabetes Mellitus with Offspring Neurodevelopment at 1 Year

**DOI:** 10.3390/nu17020258

**Published:** 2025-01-11

**Authors:** Yueqin Zhou, Xiaoyan Chen, Tianze Li, Pingming Gao, Saijun Huang, Xiaotong Wang, Zongyu Lin, Fenglian Huang, Lewei Zhu, Yeling Lu, Yanna Zhu

**Affiliations:** 1 Department of Maternal and Child Health, School of Public Health, Sun Yat-sen University, Guangzhou 510080, China; zhouyq39@mail2.sysu.edu.cn (Y.Z.); chenxy727@mail2.sysu.edu.cn (X.C.); litz5@mail2.sysu.edu.cn (T.L.); wangxt27@mail2.sysu.edu.cn (X.W.); linzy35@mail2.sysu.edu.cn (Z.L.); huangflian@mail2.sysu.edu.cn (F.H.); zhulw6@mail2.sysu.edu.cn (L.Z.); luyling7@mail2.sysu.edu.cn (Y.L.); 2 Department of Neonatology, Foshan Women and Children Hospital, Foshan 528000, China; fsgaopm666@126.com; 3 Department of Child Healthcare, Foshan Women and Children Hospital, Foshan 528000, China; tufanqie@163.com

**Keywords:** gestational diabetes, metabolite, neurodevelopment, offspring, mediate, predict

## Abstract

**Background/Objectives**: We aimed to identify neonatal circulating metabolic alterations associated with maternal gestational diabetes mellitus (GDM) and to explore whether these altered metabolites could mediate the association of GDM with offspring neurodevelopment. Additionally, we investigated whether neonatal circulating metabolites could improve the prediction of offspring neurodevelopmental disorders over traditional risk factors. **Methods**: The retrospective cohort study enrolled 1228 mother–child dyads in South China. GDM was diagnosed at 24–28 weeks of gestation. Neonatal circulating amino acids and lipid metabolites (carnitines) were measured from newborn heel blood 3–7 days postpartum. Offspring neurodevelopment was assessed at age 1 year using the Children Neuropsychological and Behavioral Examination Scale. Neurodevelopmental disorders were defined as developmental delay in any domain of the scale. **Results**: Twenty-one metabolites associated with GDM were identified, consisting of seven amino acids and fourteen carnitines. Among these metabolites, five (glycine, myristicylcarnitine, palmitoylcarnitine, octadecadienoylcarnitine, and 3-hydroxypalmitylcarnitine) mediated the negative association of GDM with offspring neurodevelopment at 1 year (mediation proportions: 3.91–10.66%). Furthermore, six metabolites (glycine, methionine, malonylcarnitine, isovalerylcarnitine, palmitoylcarnitine, and octadecadienoylcarnitine) significantly increased the predictive performance for offspring neurodevelopmental disorders at 1 year over five traditional risk factors including GDM, parity, infant sex, birth weight, and feeding patterns (area under curve: 0.762 vs. 0.718, *p* = 0.012). **Conclusions**: GDM was associated with a variety of amino acid and lipid metabolic alterations in neonatal circulation, among which certain metabolites mediated the association of GDM with adverse neurodevelopmental outcomes in offspring. Moreover, some neonatal circulating metabolites may serve as potential biomarkers that improved the prediction of offspring neurodevelopmental disorders over GDM and other traditional risk factors.

## 1. Introduction

Gestational diabetes mellitus (GDM), a common complication during pregnancy, has emerged as a significant public health concern due to its detrimental effects on both mothers and children. Women with GDM face increased risks of adverse delivery outcomes and postpartum diseases, such as abortion, stillbirth, and postpartum type 2 diabetes. Concurrently, their offspring are more susceptible to macrosomia, intrauterine distress, and other unfavorable fetal outcomes [1]. Importantly, mid- to late-pregnancy is a critical period for fetal brain development. An intrauterine hyperglycemic environment during this period can hinder short- and long-term neurodevelopment in offspring [2]. Accumulating studies have revealed that GDM contributes to offspring neurodevelopmental deficits across various domains, including language, cognition, attention, intelligence, motor, and social skills [2,3,4,5]. These deficits can manifest even at elevated maternal glucose levels below the diagnostic threshold of GDM [5]. Moreover, GDM is associated with higher risks of offspring neurological diseases such as autism spectrum disorder (ASD), attention deficit hyperactivity disorder (ADHD), and specific learning difficulties [2,6,7,8]. However, the underlying mechanisms linking GDM to adverse neurodevelopmental outcomes in offspring remain controversial [2].

GDM may cause disruptions in both maternal and fetal metabolome, among which amino acids and lipids are the most commonly disrupted metabolites [9]. The lipid metabolite carnitine, as an intermediate ester of fatty acid, has also been implicated in diabetes and insulin resistance [10]. Previous studies have shown that women with GDM exhibit altered amino acid and carnitine profiles in their blood and urine compared to those with normal pregnancies [11,12,13]. Additionally, the offspring of mothers with GDM have demonstrated similar metabolite changes in the placenta [14], meconium [15], and cord blood samples [16]. However, these studies primarily focused on maternal or fetal metabolic alterations during pregnancy, with few directly examining GDM-related metabolite changes in neonatal blood circulation. In parallel, altered amino acid and carnitine profiles have been linked to child neurodevelopment. Numerous studies have indicated that amino acid and carnitine levels in children with neurological diseases, such as ASD and cerebral palsy, differ from those in typical developing children [17,18,19,20,21,22]. Furthermore, some research has reported that altered amino acid and carnitine levels during pregnancy or childhood are associated with future neurodevelopmental outcomes [23,24]. Nevertheless, it remains unclear whether neonatal circulating metabolites are related to subsequent neurodevelopmental risks.

Given that metabolic alterations are closely related to both GDM and neurodevelopment, changes in metabolites may be important intermediate conditions in the associations of GDM with adverse neurodevelopmental outcomes in offspring. Notably, a recent animal study demonstrated that GDM disrupted metabolites in fetal mice brains and further affected hippocampal DNA methylation and gene regulation involved in cognition, thus representing a potential biological mechanism for the detrimental neurocognitive effects of GDM on offspring [25]. However, studies in humans investigating the mediating roles of neonatal circulating metabolites in the relationship between GDM and offspring neurodevelopment are remarkably sparse.

Emerging studies have revealed that various sociodemographic and clinical risk factors may serve as predictors for adverse neurodevelopmental outcomes in infants. These factors include maternal age [26], parental education [27], mode of delivery [28], parity [29], infant sex [27], birth weight [30], birth head circumference [31], gestational age [32], and feeding patterns [33]. The negative association observed between GDM and offspring neurodevelopment also suggests that GDM may strongly predict neurodevelopmental disorders in offspring. A recent birth cohort study further unveiled that an array of cord serum and infant stool metabolites, as well as multiple traditional risk factors, were associated with future neurodevelopmental disorders diagnoses [34]. These findings laid a foundation for combining metabolites with traditional risk factors such as GDM to predict future neurodevelopmental disorders in infants. Nonetheless, few studies have developed prediction models for infant neurodevelopmental disorders or explored whether adding neonatal circulating metabolites into the model would enhance its predictive performance over traditional risk factors.

Accordingly, we aimed to identify a metabolic alteration in neonatal circulation that is associated with maternal GDM. We also explored whether these altered metabolites could mediate the association of GDM with offspring neurodevelopment or improve the prediction of offspring neurodevelopmental disorders over traditional risk factors.

## 2. Materials and Methods

### 2.1. Study Design and Population

The retrospective cohort study utilized approximately 30,000 electronic health records between July 2017 and June 2020 sourced from the Foshan Women and Children Hospital, a tertiary-A hospital located in South China. The population selection process is outlined in Figure S1. Briefly, data from 4356 mother–child dyads with singleton live births were included. The infants underwent neonatal metabolite screening 3–7 days postpartum and neurodevelopmental assessment at 12 months of age. Mothers diagnosed with diabetes, polycystic ovarian syndromes, hypertension, or thyroid diseases before conception were excluded (*n* = 165). Infants with neonatal asphyxia, multiple malformations, severe congenital heart diseases, or a family history of mental disorders were also excluded (*n* = 52). Subsequently, each GDM mother with her offspring was matched with one randomly selected non-GDM control based on maternal age (±2 years) and infant sex. Eventually, a total of 1228 mother–child dyads were included for analysis, consisting of 614 GDM cases (GDM group) and 614 controls (non-GDM group as reference).

### 2.2. Diagnosis of GDM

Participating mothers underwent a 75 g oral glucose tolerance test (OGTT) at 24–28 weeks of gestation after an overnight fast of at least 8 h. Diagnosis of GDM was based on the criteria established by the International Association of Diabetes and Pregnancy Study Group (IADPSG) in 2016, which stipulates either fasting plasma glucose of ≥5.1 mmol/L, 1 h postprandial glucose of ≥10.0 mmol/L, or 2 h postprandial glucose of ≥8.5 mmol/L [35].

### 2.3. Measurements of Neonatal Circulating Metabolites

Neonatal circulating metabolites were measured from newborn heel blood samples collected 3–7 days postpartum by trained nurses. The heel blood was processed into filter paper dry blood spots, and 58 metabolites were analyzed via a targeted metabolomics approach (Table S1). Specifically, 23 amino acids and 35 carnitines were quantified using liquid chromatography-tandem mass spectrometry (LC-MS/MS) with tandem mass spectrometers (SCIEX API3200MD, Danaher Corporation, Framingham, MA, USA) and assay kits (Beijing Genomics Institute, Shenzhen, China). This panel of 58 metabolites is routinely measured for each newborn at the Foshan Women and Children Hospital as part of neonatal hereditary metabolic disease screening. All procedures were carried out strictly in accordance with the technical specifications for neonatal disease screening.

### 2.4. Assessment of Offspring Neurodevelopment

Offspring neurodevelopment was assessed at 12 months of age by professionals using the Children Neuropsychological and Behavioral Examination Scale-Revision 2016 (CNBS-R2016), which was independently compiled by the Capital Institute of Pediatric in China [36]. This scale, applicable for children aged 0–6 years, possesses considerable clinical utility in China and covers developmental milestones across different age groups. It encompasses five domains: gross motor, fine motor, adaptive behavior, language, and personal society development. The score of each domain represents the mental age. The development quotient (DQ) of each domain is calculated as the mental age divided by actual months of age and multiplied by 100, with a higher DQ indicating more favorable neurodevelopmental status. The general quotient (GQ) is the average DQ of five domains. A DQ < 70 indicates a developmental delay in that domian, and the presence of developmental delay in any domain is defined as neurodevelopmental disorders.

### 2.5. Measurements of Covariates

Maternal covariates such as age, education level, history of diseases before and during pregnancy, gravidity, parity, method of conception, and mode of delivery were obtained from medical records. Neonatal covariates, including sex, gestational age, birth weight, birth length, birth head circumference, and neonatal diseases history, were also retrieved from medical records. Feeding patterns and daily duration of outdoor activities in offspring were assessed at 12 months of age using a structured questionnaire completed by the guardians.

### 2.6. Statistical Analysis

Metabolite data were log(e)-transformed to approximate a normal distribution and standardized as Z-scores (mean = 0, SD = 1) before analysis. To identify neonatal circulating metabolic alterations associated with GDM, Student’s *t*-test and partial least squares discriminant analysis (PLS-DA) were performed. The metabolite with a *p*-value of <0.05 in Student’s *t*-test and a variance importance projection (VIP) > 1.0 in the PLS-DA was identified as differentially expressed between GDM and non-GDM groups. The associations of GDM with differentially expressed metabolites were further assessed using multiple linear regression models.

To explore whether neonatal circulating metabolites mediated the relationship between GDM and offspring neurodevelopment at 1 year, multiple linear regression models were employed to evaluate the associations of both metabolites and GDM with offspring neurodevelopment. Then, simple and multiple parallel mediation analyses were applied to examine the mediation effects of metabolites between GDM and offspring neurodevelopment. The significance of direct effect, indirect effect, and total effect were calculated using the bootstrap method with 1000 resamples. The mediation effect was deemed significant if the 95% confidence interval (CI) of the indirect effect excluded zero. The proportion mediated (%) was calculated as the percentage of the indirect effect relative to the total effect. All multiple linear regression models and mediation analyses were adjusted for a priori selected covariates, including maternal age, education level, parity, method of conception, and mode of delivery, as well as infant sex, gestational age, birth weight, birth head circumference, feeding patterns, and daily duration of outdoor activities.

To evaluate whether neonatal circulating metabolites improved the prediction of offspring neurodevelopmental disorders at 1 year over traditional risk factors, three prediction models were developed and validated. First, the metabolites associated with GDM and 12 traditional risk factors (the aforementioned covariates and GDM) were regarded as candidate predictors for offspring neurodevelopmental disorders. Second, a 10-fold cross-validation Lasso regression analysis was performed to select the GDM-associated metabolites that also showed significant associations with offspring neurodevelopmental disorders, while bidirectional stepwise logistic regression analysis was conducted to select the traditional risk factors. Third, three prediction models were developed: (1) metabolite model, including the metabolites selected by the Lasso regression; (2) traditional model, based on the traditional risk factors retained in the stepwise regression; (3) combined model, integrating both metabolites and traditional risk factors. Fourth, predictive performance of the three established models was compared in terms of discrimination, reclassification, calibration, and clinical usefulness. Discrimination was determined by calculating the area under the receiver operating characteristic (ROC) curve (AUC) for each model and comparing the differences between AUCs using the Delong test. Model discrimination was also assessed by the integrated discrimination improvement (IDI). The models’ abilities to reclassify the offspring into high- or low-risk of neurodevelopmental disorders were compared using the continuous net reclassification improvement (NRI). Calibration was evaluated using the calibration curve and Hosmer–Lemeshow test, and clinical usefulness was assessed by decision curve analysis (DCA). Internal validation was performed using the bootstrap method with 1000 resamples. Finally, a visualized nomogram was constructed to graphically present the combined model.

Statistical analyses were conducted using MetaboAnalyst version 4.0 and R version 4.3.0. A two-sided *p*-value of <0.05 was considered statistically significant.

## 3. Results

### 3.1. Characteristics of Study Population

Table 1 summarizes the maternal and offspring characteristics between GDM and non-GDM groups. The mean age of mothers at delivery was 31.2 ± 4.8 years. Among the offspring, 56.8% were male and the median gestational age at birth was 38.9 (38.1–39.7) weeks. The multiparous rate, natural conception rate, and gestational age were significantly lower in the GDM group compared to the non-GDM controls (*p* < 0.05), whereas other characteristics did not show significant differences across groups. Notably, the overall prevalence of offspring neurodevelopmental disorders at 1 year was 5.46%, with a significantly higher prevalence observed in GDM group than in controls (7.65% vs. 3.26%, *p* < 0.05).

### 3.2. Alterations in Neonatal Circulating Metabolites That Associated with Maternal GDM

The supervised 2-D PLS-DA score plot indicated a partial separation between GDM and non-GDM groups (Figure S2a), suggesting the presence of differentially expressed metabolites across groups. Figure S2b presents the metabolites with VIP > 1.0. In total, 21 neonatal circulating metabolites were identified as differentially expressed across groups, including seven amino acids and 14 carnitines (*p* < 0.05 in Student’s-*t* test and VIP > 1.0 in PLS-DA; Table 2). Specifically, three amino acids (homocysteine, methionine, and threonine) and six carnitines (free carnitine [C0], propionylcarnitine [C3], butyrylcarnitine [C4], Isovalerylcarnitine [C5], 3-hydroxyisovalerylcarnitine [C5-OH]), and octadecadienoylcarnitine [C18:2]) exhibited higher levels in GDM groups compared to non-GDM controls. Conversely, four amino acids (glutamine, glycine, lysine, and pipercide) and eight carnitines (malonylcarnitine [C3DC], glutarylcarnitine [C5DC], capryloylcarnitine [C8], laurylcarnitine [C12], myristicylcarnitine [C14:1], palmitoylcarnitine [C16], 3-hydroxypalmitylcarnitine [C16-OH], and carbamate [C24]) showed lower levels in GDM groups than in controls. After adjusting for selected covariates, 19 metabolites remained significantly associated with GDM (*p* < 0.05 in multiple linear regression; Table 2). However, the associations of GDM with methionine and C5 were attenuated after multiple adjustments.

### 3.3. Relationships Between Neonatal Circulating Metabolites and Offspring Neurodevelopment at 1 Year

**Figure 1:**
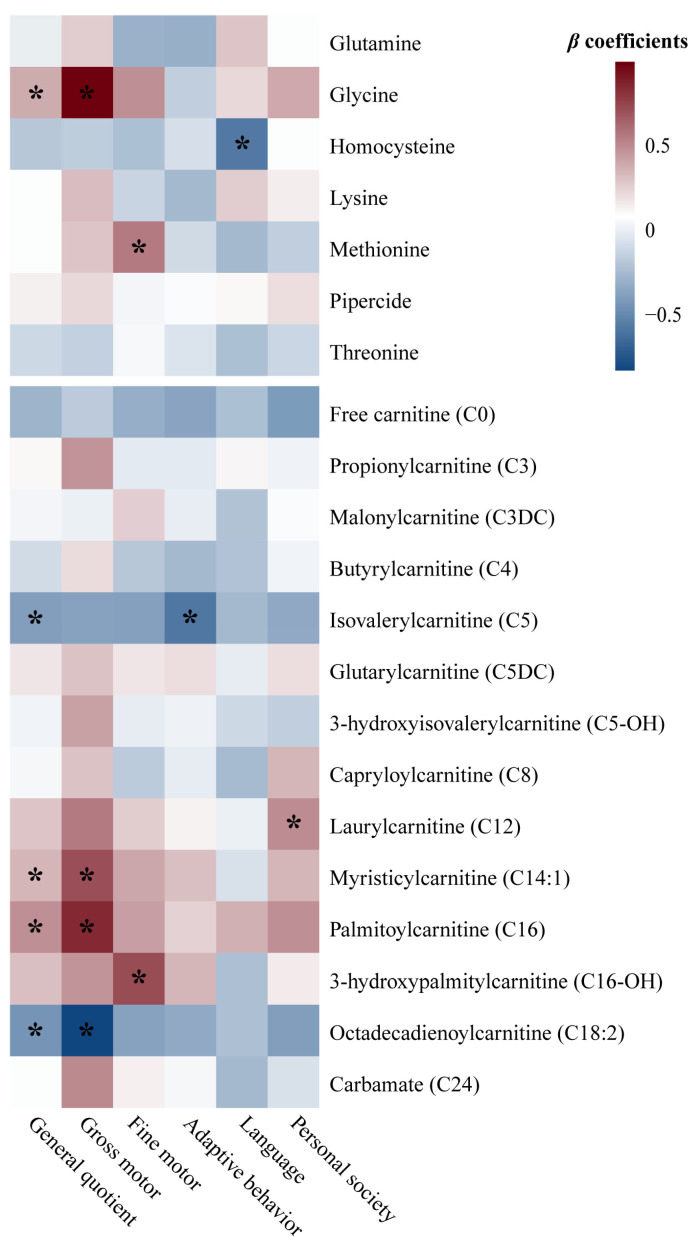
Heatmap of *β* coefficients between GDM-associated neonatal circulating metabolites and offspring neurodevelopment at 1 year, calculated by multiple linear regression analysis. * *p*-values of the *β* regression coefficients < 0.05. Metabolite data are log(e)-transformed to approximate a normal distribution and standardized as Z-scores before analysis. The *β* values express the changes in general quotient or development quotient in corresponding domain as assessed by the Children Neuropsychological and Behavioral Examination Scale-Revision 2016 that associated with one-unit increase in Z-score of log(e)-transformed metabolite levels, adjusted for maternal age, education level, parity, method of conception, and mode of delivery, as well as infant sex, gestational age, birth weight, birth head circumference, feeding patterns, and daily duration of outdoor activities. Abbreviations: GDM—gestational diabetes mellitus.

As presented in Figure 1, nine GDM-associated neonatal circulating metabolites also showed significant associations with offspring neurodevelopment at 1 year after adjusting for potential confounders, including three amino acids and six carnitines (*p* < 0.05). In detail, glycine, C14:1, and C16 were positively associated, while C5 and C18:2 were negatively associated with GQ. Glycine, C14:1, and C16 exhibited positive associations, whereas C18:2 displayed a negative association with gross motor development. Methionine and C16-OH were positively associated with fine motor development. C5 was inversely associated with adaptive behavior development. Homocysteine was inversely associated with language development. C12 showed a positive association with personal society development.

### 3.4. Mediation Effects of Neonatal Circulating Metabolites in the Relationship Between Maternal GDM and Offspring Neurodevelopment at 1 Year

**Figure 2:**
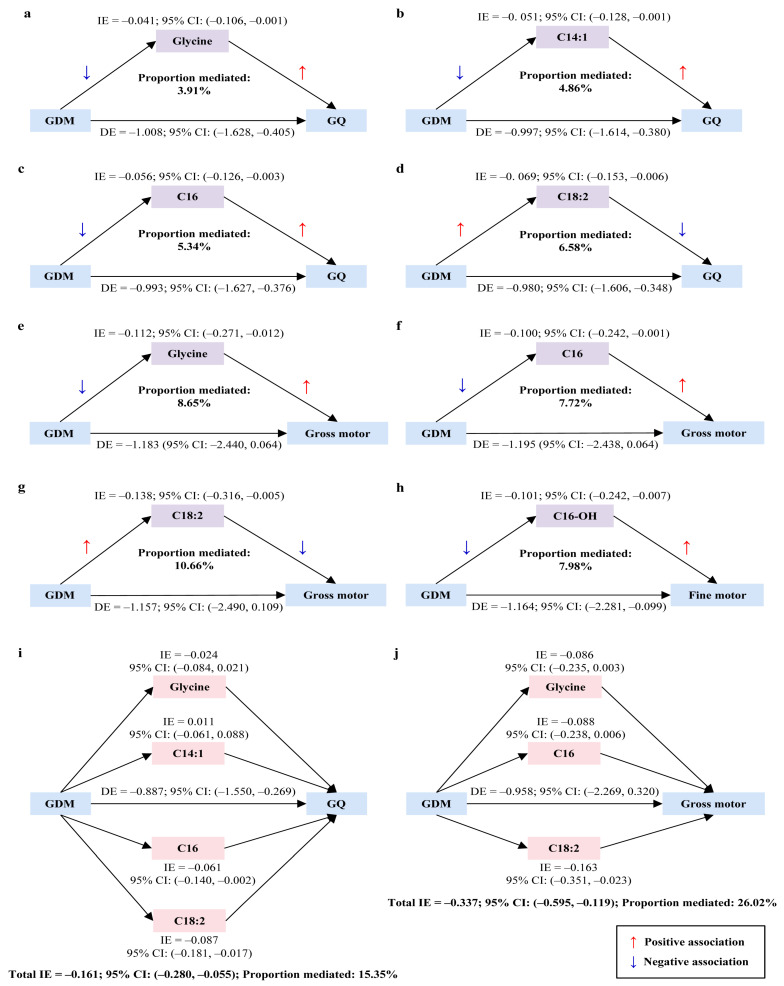
Mediation effects of neonatal circulating metabolites in the association of maternal GDM with offspring neurodevelopment at 1 year. (**a**–**h**) Simple mediation effects. (**i**,**j**) Multiple parallel mediation effects. Metabolite data are log(e)-transformed to approximate a normal distribution and standardized as Z-scores before analysis. Mediation analysis is adjusted for maternal age, education level, parity, method of conception, and mode of delivery, as well as infant sex, gestational age, birth weight, birth head circumference, feeding patterns, and daily duration of outdoor activities. Part of the TE of GDM on offspring neurodevelopment may operate through the metabolites, referred to as the IE. The remaining part of the TE, not explained by metabolites and representing all the other possible explanations, is referred to as the DE. The mediation effect is deemed significant if the 95% CI of IE excludes zero. The proportion mediated (%) is calculated as the percentage of IE relative to the TE. Abbreviations: IE—indirect effect; CI—confidence interval; GDM—gestational diabetes mellitus; GQ—general quotient; DE—direct effect; TE—total effect.

GDM was significantly and negatively associated with offspring neurodevelopment at 1 year even after adjustment for covariates, specifically associated with GQ and gross motor, fine motor, and adaptive behavior development (*p* < 0.05; Table S2). In these associations, five neonatal circulating metabolites (glycine, C14:1, C16, C16-OH, and C18:2) exhibited significant mediation effects. Simple mediation analysis indicated that glycine, C14:1, C16 and C18:2 partially mediated the association between GDM and GQ (proportions mediated: 3.91%, 4.86%, 5.34%, and 6.58%, respectively; Figure 2a–d). Glycine, C16, and C18:2 were found fully mediated the association between GDM and gross motor development (proportions mediated: 8.65%, 7.72%, and 10.66%, respectively; Figure 2e–g). C16-OH served as a partial mediator between GDM and fine motor development (proportion mediated: 7.98%; Figure 2h). The further multiple parallel mediation analysis revealed that the four metabolites (glycine, C14:1, C16, and C18:2) collectively mediated 15.35% of the association between GDM and GQ as a partial mediator. When considering each metabolite individually, the mediation effects of C16 and C18:2 remained significant, but no significant effects were observed for glycine and C14:1 (Figure 2i). Moreover, the three metabolites (glycine, C16, and C18:2) acted as a full mediator and jointly accounted for 26.02% of the association between GDM and gross motor development. However, only C18:2 maintained a significant mediation effect when examined individually, whereas glycine and C16 did not (Figure 2j).

### 3.5. Predictive Performance of Neonatal Circulating Metabolites for Offspring Neurodevelopmental Disorders at 1 Year Compared to Traditional Risk Factors

**Figure 3:**
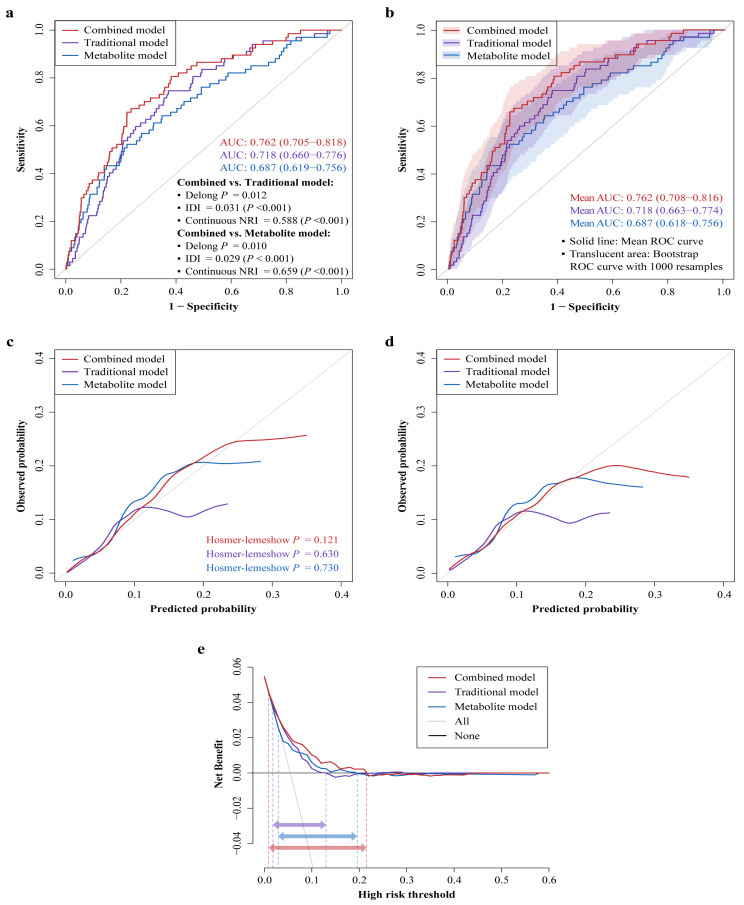

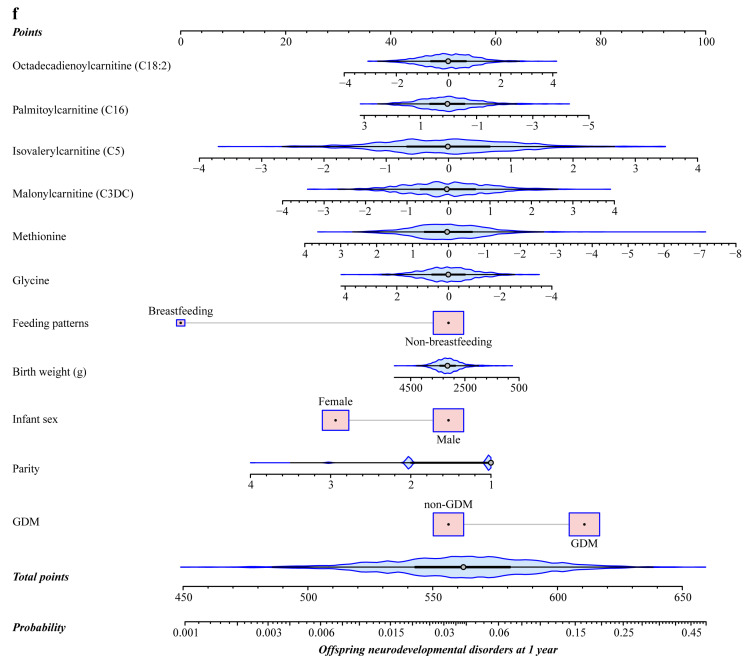
Predictive performance of neonatal circulating metabolites for offspring neurodevelopmental disorders at 1 year compared to traditional risk factors. (**a**,**b**) ROC curves comparing the combined, traditional, and metabolite models. (**a**) Shows the ROC curve and AUC (95% CI) of each model, and *p*-values of the Delong test, IDIs, and continuous NRIs for combined vs. traditional models and combined vs. metabolite models. (**b**) Depicts the ROC curves from internal validation using the bootstrap method (1000 resamples) and the mean AUCs (95% CIs). (**c**,**d**) Calibration curves for the three prediction models. The ideal curve is the 45° line, which indicates perfect prediction. *p* > 0.05 in Hosmer–Lemeshow test suggests no significant difference in goodness-of-fit between predicted and observed values. (**c**) Shows the apparent calibration curves. (**d**) Displays the bias-corrected calibration curves from internal validation using the bootstrap method (1000 resamples). (**e**) DCA curves for the three prediction models, which are internally validated by the bootstrap method (1000 resamples). The red, purple, and blue horizontal arrows indicate the threshold probability ranges of combined, traditional, and metabolite models, respectively. (**f**) Nomogram depicting the combined model for predicting offspring neurodevelopmental disorders at 1 year (see Figure S5 for detailed usage of the nomogram). Metabolite data are log(e)-transformed to approximate normal distribution and calculated as Z-scores to standardize before analysis. Abbreviations: AUC—area under curve; IDI—integrated discrimination improvement; NRI—net reclassification improvement; ROC—receiver operating characteristic; GDM—gestational diabetes mellitus; CI—confidence interval; DCA—decision curve analysis.

The Lasso regression identified six GDM-associated neonatal circulating metabolites (glycine, methionine, C3DC, C5, C16, and C18:2) that also showed significant associations with offspring neurodevelopmental disorders at 1 year (Figure S3). Meanwhile, the stepwise regression retained five traditional risk factors (GDM, parity, infant sex, birth weight, and feeding patterns) that associated with offspring neurodevelopmental disorders at 1 year (Figure S4). Figure 3 presents the predictive performance of the three established models for predicting offspring neurodevelopmental disorders at 1 year. The AUC, IDI, and continuous NRI favored that the combined model showed remarkably improved discrimination and reclassification compared to either the traditional or metabolite model (Figure 3a,b). For example, the AUC increased significantly from 0.718 (95% CI: 0.660–0.776) to 0.762 (95% CI: 0.705–0.818) for combined vs. traditional models (Delong *p* = 0.012), with both the IDI (IDI = 0.031, *p* < 0.001) and continuous NRI (NRI = 0.588, *p* < 0.001) being significant positive values. The combined model was also better calibrated relative to the other two models (Figure 3c,d). The calibration curve of the combined model was closest to the ideal curve, and the Hosmer–Lemeshow test yielded a non-significant *p*-value of 0.121, suggesting no statistical deviation from a perfect fit between the predicted and observed values. The DCA curve indicated that within a threshold probability range of 1–22%, using the combined model to predict offspring neurodevelopmental disorders would add more net benefit than using either the traditional or metabolite model, suggesting that the combined model possessed superior clinical usefulness (Figure 3e). These results were still robust after internal validation using 1000 bootstrap resamples. Taken together, the prediction model combing six neonatal circulating metabolites with five traditional risk factors achieved the best performance, outperforming the models based solely on traditional risk factors or metabolites. Thus, a nomogram based on the combined model was constructed (Figure 3f), providing a convenient and personalized tool to predict the probability of offspring neurodevelopmental disorders at 1 year. A detailed description of the nomogram’s usage can be found in Figure S5.

## 4. Discussion

In the present study, we identified 21 neonatal circulating metabolites associated with maternal GDM, including seven amino acids and 14 carnitines. Among these metabolites, nine (glycine, homocysteine, methionine, C5, C12, C14:1, C16, C16-OH, C18:2) exhibited significant associations with offspring neurodevelopment at 1 year, and five (glycine, C14:1, C16, C16-OH, and C18:2) mediated the negative association of GDM with offspring neurodevelopment at 1 year. Moreover, six neonatal circulating metabolites (glycine, methionine, C3DC, C5, C16, and C18:2) significantly increased the predictive performance for offspring neurodevelopmental disorders at 1 year over five traditional risk factors including GDM, parity, infant sex, birth weight, and feeding patterns.

Our study indicated that GDM was associated with an altered metabolite profile in newborn heel blood, characterized by seven amino acids and 14 carnitines. These results are similar to some previous studies using different biological samples [11,12,13,14,15,16]. However, our findings may provide a more representative insight into GDM-induced metabolic alterations in neonatal circulation. The precise mechanisms underlying these changes remain uncertain, possibly resulting from altered placental transport of metabolites, or as secondary consequences of hyperglycemia and compensatory insulin secretion in the fetus [37,38]. Notably, carnitine metabolism may be particularly susceptible when GDM disrupts glycolipid and amino acid metabolism in offspring. Carnitine can be synthesized from two essential amino acids and is involved in the *β*-oxidation process of fatty acids [10]. Long-chain fatty acids are transported from the cytoplasm into the mitochondrial matrix by the carnitine shuttle and then converted to long-chain acylcarnitines via carnitine palmitoyltransferase-1 (CTP-1) located in the mitochondrial outer membrane [10,39]. In our study, several glucogenic (e.g., glutamine, glycine, methionine, and threonine) and ketogenic (e.g., lysine) amino acids were differentially expressed in GDM offspring compared to non-GDM controls, aligning with studies linking these amino acids to GDM, type 2 diabetes, or insulin resistance [40,41]. However, we observed no significant associations between GDM and aromatic (e.g., tryrosine, phenylalanine, and tryptophan) or branched-chain (e.g., leucine, isoleucine, and valcine) amino acids (BCAAs), contrary to evidence linking these amino acids to present or future diabetes [40,41]. To some extent, our observation that GDM offspring had elevated levels of BCAA-derived C3- and C5-carnitines was in accordance with this evidence [39]. Also, we found that the levels of most medium- (6–12 carbons) and long-chain (14 and more carbons) acylcarnitines were lower in GDM offspring than in controls. Likewise, lipidomics research has unveiled the strong impact of GDM on placental acylcarnitine profiles, particularly reduction in medium- and long-chain acylcarnitines, which suggests mitochondrial dysfunction due to CTP-1 shuttling restriction [14]. Alternatively, GDM offspring may have received sufficient maternal glucose during fetal life as primary energy sources and thus reduced their own fat mobilization, in which medium- and long-chain fatty acids may serve as the main mobilizing substances [42]. Given that C0 is crucial for transporting long-chain fatty acids into the mitochondria, the higher C0 levels observed in GDM offspring may result from decreased long-chain fatty acid mobilization. Taken together, our findings suggested that the neonates born to mothers with GDM had unfavorable amino acid and lipid metabolic profiles in their blood circulation.

In our study, certain GDM-associated neonatal circulating metabolites also showed significant associations with offspring neurodevelopment at 1 year. Among the amino acids, glycine and methionine were positively associated, whereas homocysteine was negatively associated with offspring neurodevelopment. Glycine has been proven to precisely regulate hippocampal functions, such as learning and memory, by balancing *N*-methyl-D-aspartic acid receptor (NMDAR)-dependent neuronal excitation and glycine receptor (GlyR)-mediated GABAergic inhibition [43]. Methionine and homocysteine can interconvert via the methionine cycle. Methionine is a precursor of cellular methylation and has been recognized for its neuroprotective functions [44]. On the contrary, homocysteine is neurotoxic and hyperhomocysteinemia has been reported as a risk factor for multiple neuropsychiatric disorders [45]. In addition, our study revealed that several short- (2–5 carbons) and medium-chain (6–12 carbons) acylcarnitines, as well as various long-chain (14 and more carbons) acylcarnitines, were significantly associated with offspring neurodevelopment. To date, the relationships between carnitines and neurodevelopment remain understudied and inconclusive. A Chinese study observed lower levels of C0, and short- and long-chain acylcarnitine in children with ASD compared to typically developing children [21]. Conversely, another large cohort study found elevated levels of short- and long-chain but not medium-chain acylcarnitine in children with ASD [22]. Given the unique neuroprotective, neuromodulatory, and neurotrophic properties of carnitines [46], the impact of disrupted carnitine metabolism on infant neurodevelopment warrants further investigation.

Our findings that five neonatal circulating metabolites, including glycine and four long-chain acylcarnitines, mediated the negative association of GDM with offspring neurodevelopment at 1 year provided clues for the biological mechanisms linking GDM to adverse neurodevelopmental outcomes in offspring. GDM may negatively impact offspring neurodevelopment by lowering neonatal circulating glycine levels. The mechanisms involving GDM-induced reduction in glycine may be multifaceted. Since glycine is a glucogenic amino acid, increased gluconeogenesis related to diabetes may promote glycine consumption [47]. Additionally, glycine can conjugate with acyl groups from β-oxidation intermediates (e.g., acyl-CoA esters) or BCAA catabolism byproducts. Thus, excessive fatty acyl group formation and elevated BCAAs related to diabetes may enhance glycine utilization and excretion [47,48]. Furthermore, persistent oxidative stress induced by diabetes may accelerate the synthesis of antioxidants like glutathione, resulting in the depletion of glycine substrates [47]. These processes may collectively disrupt the delicate balance of neural excitation and inhibition regulated by glycine, thereby leading to neurodevelopmental impairments. Our results also suggested that GDM may adversely affect offspring neurodevelopment by altering neonatal circulating long-chain acylcarnitine profiles, namely by reducing C14:1, C16, and C16-OH levels while increasing C18:2 levels. The precise mechanisms underlying these alterations remain elusive. As aforementioned, decreased long-chain acylcarnitines related to diabetes may reflect limitations in β-oxidation flux at the CTP-1 step [14]. Conversely, elevated long-chain acylcarnitines may indicate incomplete and defective mitochondrial fatty acid oxidation (FAO), as seen in some patients with diabetes or insulin resistance. In such cases, FAO exceeds the capacity of the tricarboxylic acid cycle and electron transport chain, leading to the accumulation of FAO intermediates such as acylcarnitines [39,49]. Both scenarios imply mitochondrial dysfunction, which may impair brain energy metabolism and precipitate neurodegenerative pathologies [46].

From a clinical perspective, it is crucial to identify high-risk individuals before the onset of neurodevelopmental disorders. Therefore, we developed a prediction model for offspring neurodevelopmental disorders at 1 year that combined six neonatal circulating metabolites with five traditional risk factors. Our results demonstrated that adding these metabolites into the model significantly enhanced its predictive performance over traditional risk factors including GDM, suggesting that these metabolites may serve as potential biomarkers for early identifying offspring neurodevelopmental disorders. Our findings implied that glycine, methionine, and C16 may act as negative predictors for the risks of offspring neurodevelopmental disorders, while C3DC, C5, and C18:2 seemed to be positive predictors. Glycine and methionine have been recognized for their neuroprotective roles [43,44]. The potential adverse neurodevelopmental effects of C3DC and C5, as BCAA-derived carnitines, may result from the neurotoxicity from BCAAs [50]. Decreased levels of long-chain acylcarnitine, such as C16, may signify limited mitochondrial *β*-oxidation during brain energy metabolism. In contrast, increased levels of long-chain acylcarnitine, such as C18:2, may reflect incomplete mitochondrial FAO. Among the five traditional risk factors retained in the prediction model, GDM, primiparity, male sex, lower birth weight, and non-breastfeeding were predictors for heightened risks of offspring neurodevelopmental disorders, consistent with existing evidence [2,27,29,30,33]. Collectively, our findings suggested that combining representative neonatal circulating amino acids and lipid metabolites with traditional risk factors including GDM may better predict neurodevelopmental disorders in offspring.

Once newborns at high risk of developing neurodevelopmental disorders are identified, prevention becomes a critical issue in clinical practice. It is particularly important for those born to mothers with GDM, as GDM is a strong predictor for poor neurodevelopmental outcomes in offspring. Our findings provide clues on early prevention strategies for child neurodevelopmental disorders. From the perspective of modifiable traditional risk factors, controlling maternal glucose levels during pregnancy and advocating breastfeeding postpartum may effectively mitigate the risks of future neurodevelopmental disorders in offspring. Additionally, our study highlights that metabolic disruptions associated with GDM, specifically reduced levels of neonatal circulating glycine and long-chain acylcarnitines, may impair future neurodevelopment. These metabolites may serve as targeted biomarkers for preventing neurodevelopmental disorders in offspring of mothers with GDM, or for attenuating detrimental effects of GDM on offspring neurodevelopment. Acylcarnitines are downstream metabolites of fatty acids, and body levels of amino acids and fatty acids can be regulated by diets and nutritional supplements [51]. Therefore, immediate actions taken after birth may include monitoring circulating metabolites levels to identify changes in key metabolites and assess future risks of neurodevelopmental disorders. For high-risk newborns, formula fortified with glycine and long-chain fatty acids could be considered. Alternatively, increasing the intake of glycine- and long-chain fatty acid-rich foods in postnatal diets of the mothers could also provide these nutrients to offspring through breastfeeding, which may be a more convenient and feasible way. Furthermore, early neurodevelopmental support, such as cognitive and motor skill stimulation, could be considered to mitigate neurodevelopmental delays. However, the reversibility of neurodevelopmental impairments resulting from GDM-induced neonatal metabolic alterations remains uncertain. A recent animal study has shown that systemic serine deficiency and dyslipidemia can drive diabetic peripheral neuropathy. Normalization of serine levels by dietary supplementation and mitigation of dyslipidemia with myristic acid can alleviate neuropathy in diabetic mice [52]. These findings support our hypothesis that interventions targeting specific amino acids and lipids may attenuate GDM-induced neurodevelopmental impairments in offspring. However, further research is still warranted to determine whether these impairments can be fully reversed or simply alleviated. Moreover, since metabolites can be inter-converted in vivo, further animal experiments and intervention studies are essential to explore optimal intervention timing and appropriate supplemental dosage before actual practical application.

Our study had several strengths. Firstly, we directly measured metabolites in newborn heel blood and unveiled neonatal circulating metabolic alterations that associated with GDM. Furthermore, to our knowledge, this is the first study investigating the mediating roles of neonatal circulating metabolites in the association of GDM with offspring neurodevelopment and exploring the predictive performance of these metabolites for offspring neurodevelopmental disorders. Nonetheless, some limitations should be acknowledged. First, the study population derived from a single hospital limits generalizability of our findings. Second, due to the retrospective nature of our study, certain potential confounders not documented or with substantial missing values in medical records were not adjusted for, such as maternal pre-pregnancy obesity, gestational weight gain, and management of GDM (e.g., lifestyle interventions including dietary control and exercise, as well as medical therapy including insulin and metformin), which may cause residual confounding. Third, the lack of documented maternal blood glucose levels in medical records restricted our ability to assess possible effects of different GDM severities on neonatal circulating metabolic profiles and offspring neurodevelopment through stratified analyses by glucose levels. Fourth, our predictive analyses were conducted on the same dataset. Although internal validation using 1000 bootstrap resamples has been performed, external validation through an independent longitudinal cohort is still required to confirm the robustness of our findings. Finally, owing to the nature of observational research, our findings are intrinsically exploratory and cannot conclusively establish causality.

## 5. Conclusions

In conclusion, maternal GDM was associated with a variety of amino acid and lipid metabolic alterations in neonatal circulation, among which certain metabolites mediated the association of GDM with adverse neurodevelopmental outcomes in offspring. Moreover, some neonatal circulating metabolites may serve as potential biomarkers that improved the prediction of offspring neurodevelopmental disorders over GDM and other traditional risk factors. Our findings shed light on the potential biological mechanisms underlying GDM-induced neurodevelopmental impairments in offspring and, additionally, lay a theoretical basis for the early identification and prevention of offspring neurodevelopmental disorders. However, further extensive and long-term multi-center prospective research is still warranted to validate these findings.

## Figures and Tables

**Table 1:** Characteristics of mother–child dyads between GDM and non-GDM groups.

Characteristics	Overall ^a^ *N* = 1228	GDM ^a^ *n* = 614	Non-GDM ^a^ *n* = 614	*p*-Value ^b^
Maternal characteristics				
Age at delivery, years	31.2 ± 4.8	31.2 ± 4.8	31.2 ± 4.8	0.895
Education level, *n* (%)				0.566
High-school and below	553 (45.0)	282 (45.9)	271 (44.1)	
College and above	675 (55.0)	332 (54.1)	343 (55.9)	
Gestational hypertension, *n* (%)	17 (1.4)	7 (1.1)	10 (1.6)	0.625
Thalassemia, *n* (%)	99 (8.1)	46 (7.5)	53 (8.6)	0.529
Primigravid, *n* (%)	412 (33.6)	218 (35.5)	194 (31.6)	0.165
Multiparous, *n* (%)	584 (47.6)	272 (44.3)	312 (50.8)	0.026
Natural conception, *n* (%)	1108 (90.2)	541 (88.1)	567 (92.3)	0.016
Spontaneous vaginal delivery, *n* (%)	792 (64.5)	389 (63.4)	403 (65.6)	0.438
Offspring characteristics at birth				
Male sex, *n* (%)	698 (56.8)	349 (56.8)	349 (56.8)	1.000
Gestational age, weeks	38.9 (38.1–39.7)	38.9 (38.0–39.6)	39.0 (38.1–39.9)	0.003
Birth weight, g	3103.1 ± 485.5	3082.2 ± 480.6	3124.0 ± 489.8	0.132
Birth length, cm	49.0 (48.0–50.0)	49.0 (48.0–50.0)	49.0 (48.0–50.0)	0.447
Birth head circumference, cm	32.8 ± 1.9	32.8 ± 1.8	32.7 ± 2.0	0.655
Offspring characteristics at 1 year				
Breastfeeding, *n* (%)	86 (7.0)	41 (6.7)	45 (7.3)	0.737
Duration of outdoor activities, hours/day	1.0 (0.0–1.0)	1.0 (0.0–1.0)	1.0 (0.0–1.0)	0.880
Neurodevelopmental disorders ^c^, *n* (%)	67 (5.46)	47 (7.65)	20 (3.26)	0.001

Abbreviations: GDM—gestational diabetes mellitus. ^a^ The data are presented as mean ± standard deviation, median (interquartile range), or *n* (%). ^b^ Calculated using Student’s *t*-test, Mann–Whitney *U* test, or *χ*^2^ test, compared between GDM and non-GDM groups. *p* < 0.05 indicates statistically significant. ^c^ Neurodevelopmental disorders are defined as developmental delay in any domain of the Children Neuropsychological and Behavioral Examination Scale-Revision 2016.

**Table 2:** Relationships between maternal GDM and neonatal circulating metabolites.

Metabolites	Median (IQR) ^a^		*p*-Value ^b^	VIP ^c^	*β* (95% CI) ^d^	
	GDM *n* = 614	Non-GDM *n* = 614			Crude	Adjusted ^e^
Amino acids						
Glutamine	22.614 (10.001)	23.786 (10.181)	0.039	1.050	−0.118 (−0.230, −0.006)	−0.126 (−0.238, −0.014)
Glycine	196.751 (50.194)	202.277 (50.910)	0.047	1.010	−0.113 (−0.225, −0.001)	−0.117 (−0.229, −0.005)
Homocysteine	13.670 (2.588)	13.482 (2.455)	0.041	1.040	0.117 (0.005, 0.228)	0.123 (0.010, 0.235)
Lysine	20.003 (8.522)	21.221 (8.554)	0.041	1.038	−0.116 (−0.228, −0.005)	−0.127 (−0.239, −0.014)
Methionine	27.220 (9.139)	26.683 (8.688)	0.042	1.032	0.116 (0.004, 0.228)	0.108 (−0.004, 0.220)
Pipercide	138.644 (36.911)	149.254 (40.572)	<0.001	1.904	−0.2124 (−0.325, −0.102)	−0.214 (−0.326, −0.102)
Threonine	29.807 (10.814)	28.474 (9.448)	0.001	1.638	0.184 (0.072, 0.295)	0.173 (0.063, 0.283)
Carnitines						
Free carnitine (C0)	26.389 (9.192)	25.584 (8.909)	0.010	1.304	0.146 (0.035, 0.258)	0.134 (0.023, 0.244)
Propionylcarnitine (C3)	2.068 (0.959)	1.857 (0.922)	<0.001	2.226	0.250 (0.139, 0.361)	0.254 (0.142, 0.365)
Malonylcarnitine (C3DC)	0.059 (0.023)	0.061 (0.024)	0.028	1.118	−0.125 (−0.237, −0.014)	−0.125 (−0.237, −0.013)
Butyrylcarnitine (C4)	0.246 (0.090)	0.239 (0.099)	0.031	1.095	0.123 (0.011, 0.235)	0.120 (0.008, 0.232)
Isovalerylcarnitine (C5)	0.117(0.053)	0.112(0.046)	0.034	1.078	0.121 (0.009, 0.233)	0.096 (−0.011, 0.202)
Glutarylcarnitine (C5DC)	0.081 (0.036)	0.084 (0.040)	0.017	1.218	−0.137 (−0.248, −0.025)	−0.134 (−0.246, −0.022)
3-hydroxyisovalerylcarnitine (C5-OH)	0.220 (0.081)	0.205 (0.082)	<0.001	1.878	0.211 (0.099, 0.322)	0.203 (0.092, 0.314)
Capryloylcarnitine (C8)	0.068 (0.029)	0.070 (0.030)	0.032	1.090	−0.122 (−0.234, −0.010)	−0.122 (−0.234, −0.010)
Laurylcarnitine (C12)	0.085 (0.041)	0.094 (0.047)	<0.001	2.440	−0.274 (−0.385, −0.163)	−0.247 (−0.355, −0.139)
Myristicylcarnitine (C14:1)	0.117 (0.063)	0.127 (0.066)	0.001	1.753	−0.197 (−0.308, −0.085)	−0.174 (−0.284, −0.064)
Palmitoylcarnitine (C16)	2.440 (0.969)	2.520 (1.083)	0.011	1.297	−0.145 (−0.257, −0.034)	−0.125 (−0.233, −0.016)
3-hydroxypalmitylcarnitine (C16-OH)	0.026 (0.013)	0.028 (0.012)	0.003	1.518	−0.170 (−0.282, −0.059)	−0.151 (−0.261, −0.040)
Octadecadienoylcarnitine (C18:2)	1.132 (0.574)	1.048 (0.583)	<0.001	1.839	0.206 (0.095, 0.318)	0.179 (0.072, 0.285)
Carbamate (C24)	0.061 (0.027)	0.063 (0.027)	0.010	1.311	−0.147 (−0.259, −0.035)	−0.136 (−0.248, −0.023)

Abbreviations: GDM—gestational diabetes mellitus; IQR—interquartile range; VIP—variance importance in projection; CI—confidence interval; PLS-DA—partial least squares discriminant analysis. ^a^ Describes the levels of metabolites measured in newborn heel blood. Metabolite data are log(e)-transformed to approximate a normal distribution and standardized as Z-scores before analysis. A total of 21 metabolites with *p* < 0.05 in Student’s *t*-test and VIP > 1.0 in the PLS-DA are presented in this table. ^b^ Calculated using Student’s *t*-test, compared between GDM and non-GDM groups. *p* < 0.05 indicates statistically significant. ^c^ Calculated using the PLS-DA. ^d^ *β* values express the changes in Z-score of log(e)-transformed metabolite levels that associated with GDM (non-GDM group as reference), calculated using linear regression analysis. ^e^ Adjusted for maternal age, education level, parity, method of conception, and mode of delivery, as well as infant sex, gestational age, birth weight, birth head circumference, feeding patterns, and daily duration of outdoor activities.

## Data Availability

The data presented in this study are available on request from the corresponding author due to privacy. All proposals requesting data access will need to specify how the data will be used.

## Supplementary Materials

The following supporting information can be downloaded at: https://www.mdpi.com/article/10.3390/nu17020258/s1, Table S1: Categories of circulating metabolites measured from newborn heel blood sample; Table S2: Relationship between maternal GDM and offspring neurodevelopment at 1 year; Figure S1: Selection profile of the study population; Figure S2: Comparison of neonatal circulating metabolite levels between GDM and non-GDM groups using the PLS-DA; Figure S3: Selection of GDM-associated neonatal circulating metabolites for predicting offspring neurodevelopmental disorders at 1 year using the LASSO regression analysis with tenfold cross-validation; Figure S4: Selection of traditional risk factors for predicting offspring neurodevelopmental disorders at 1 year using the bidirectional stepwise logistic regression analysis; Figure S5: Nomogram depicting the combined model for predicting offspring neurodevelopmental disorders at 1 year.
