# Supplementary material for: Neonatal Circulating Amino Acids and Lipid Metabolites Mediate the Association of Maternal Gestational Diabetes Mellitus with Offspring Neurodevelopment at 1 Year
Source: Nutrients. 2025 Jan 11;17(2):258. doi: 10.3390/nu17020258 (PMC11767549; doi:10.3390/nu17020258)
Supplement: Supplementary file 1 [file nutrients-17-00258-s001.zip › nutrients-3313288-supplementary.pdf]

Supplementary Material

Contents

Table S1. Categories of circulating metabolites measured from newborn heel blood sample.. 2

Table S2. Relationship between maternal GDM and offspring neurodevelopment at 1 year ... 4

Figure S1. Selection profile of the study population. 5

Figure S2. Comparison of neonatal circulating metabolite levels between GDM and non-GDM groups using the PLS-DA. 6

Figure S3. Selection of GDM-associated neonatal circulating metabolites for predicting offspring neurodevelopmental disorders at 1 year using the LASSO regression analysis with tenfold cross-validation. 7

Figure S4. Selection of traditional risk factors for predicting offspring neurodevelopmental disorders at 1 year using the bidirectional stepwise logistic regression analysis. 8

Figure S5. Nomogram depicting the combined model for predicting neurodevelopmental disorders at 1 year. 9

Table S1. Categories of circulating metabolites measured from newborn heel blood sample.

Metabolite categories	Name
Amino acids	Alanine
	Arginine
	Asparagine
	Aspartic acid
	Citrulline
	Cysteine
	Glutamine
	Glutamic acid
	Glycine
	Phenylalanine
	Histidine
	Leucine
	Lysine
	Methionine
	Ornithine
	Homocysteine
	Pipercide
	Proline
	Serine
	Threonine
	Tryptophan
	Tyrosine
	Valine
Carnitines	Free carnitine (C0)
	Acetylcarnitine (C2)
	Propionylcarnitine (C3)
	Malonylcarnitine (C3DC)
	Butyrylcarnitine (C4)
	3-hydroxybutyrylcarnitine (C4-OH)
	Succinylcarnitine (C4DC)
	Isovalerylcarnitine (C5)
	3-hydroxyisovalerylcarnitine (C5-OH)
	Glutarylcarnitine (C5DC)

Isopentylcarnitine (C5:1)
Hexanoylcarnitine (C6)
Adipylcarnitine (C6DC)
Capryloylcarnitine (C8)
Decanoicacidcarnitine (C10)
Decanoylcarnitine (C10:1)
Decadienoylcarnitine (C10:2)
Laurylcarnitine (C12)
Myristicylcarnitine (C14)
3-hydroxymyristicylcarnitine (C14-OH)
Nutmegdiacylcarnitine (C14DC)
Myristicylcarnitine (C14:1)
Nutmegdienoylcarnitine (C14:2)
Palmitoyl-carnitine (C16)
3-hydroxypalmitylcarnitine (C16-OH)
3-hydroxypalmitoleylcarnitine (C16:1-OH)
Octadecylcarnitine (C18)
Octadecanoylcarnitine (C18:1)
Octadecadienoylcarnitine (C18:2)
3-hydroxyoctadecanoylcarnitine (C18-OH)
3-hydroxy-octadecanoylcarnitine (C18:1-OH)
Eicosacylcarnitine (C20)
Dococarnitine (C22)
Carbamate (C24)
Hexadecanoylcarnitine (C26)

Table S2. Relationship between maternal GDM and offspring neurodevelopment at 1 year.

Neurodevelopment	Mean ± SD [a]		β (95% CI) [b]	
	GDM (n = 614)	Non-GDM (n = 614)	Crude	Adjusted [c]
General quotient	90.93 ± 6.23 [d]	92.02 ± 4.95 [d]	–1.09 (–1.72, –0.46)	–1.05 (–1.67, –0.43)
Gross motor	92.91 ± 11.89 [d]	94.30 ± 11.04 [d]	–1.39 (–2.67, –0.10)	–1.29 (–2.58, –0.01)
Fine motor	89.26 ± 9.95 [d]	90.58 ± 8.61 [d]	–1.32 (–2.37, –0.28)	–1.27 (–2.29, –0.24)
Adaptive behavior	93.11 ± 9.71 [d]	94.40 ± 9.02 [d]	–1.30 (–2.34, –0.25)	–1.23 (–2.27, –0.20)
Language	89.80 ± 9.26	90.73 ± 8.32	–0.93 (–1.92, 0.06)	–0.89 (–1.87, 0.08)
Personal society	89.58 ± 8.09	90.03 ± 7.26	–0.45 (–1.31, 0.41)	–0.50 (–1.36, 0.36)

GDM, gestational diabetes mellitus; SD, standard deviation; CI, confidence interval; CNBS-R2016, Children Neuropsychological and Behavioral Examination Scale-Revision 2016.

[a] Describes the general quotient or development quotient in corresponding domain as assessed by the CNBS-R2016.

[b] β values express the changes in general quotient or development quotient in corresponding domain as assessed by the CNBS-R2016 that associated with GDM (non-GDM group as reference), calculated using linear regression analysis.

[c] Adjusted for maternal age, education level, parity, method of conception, and mode of delivery, as well as infant sex, gestational age, birth weight, birth head circumference, feeding patterns, and daily duration of outdoor activities.

[d] $p < 0.05$ between GDM and non-GDM groups, compared using the Student's t test.

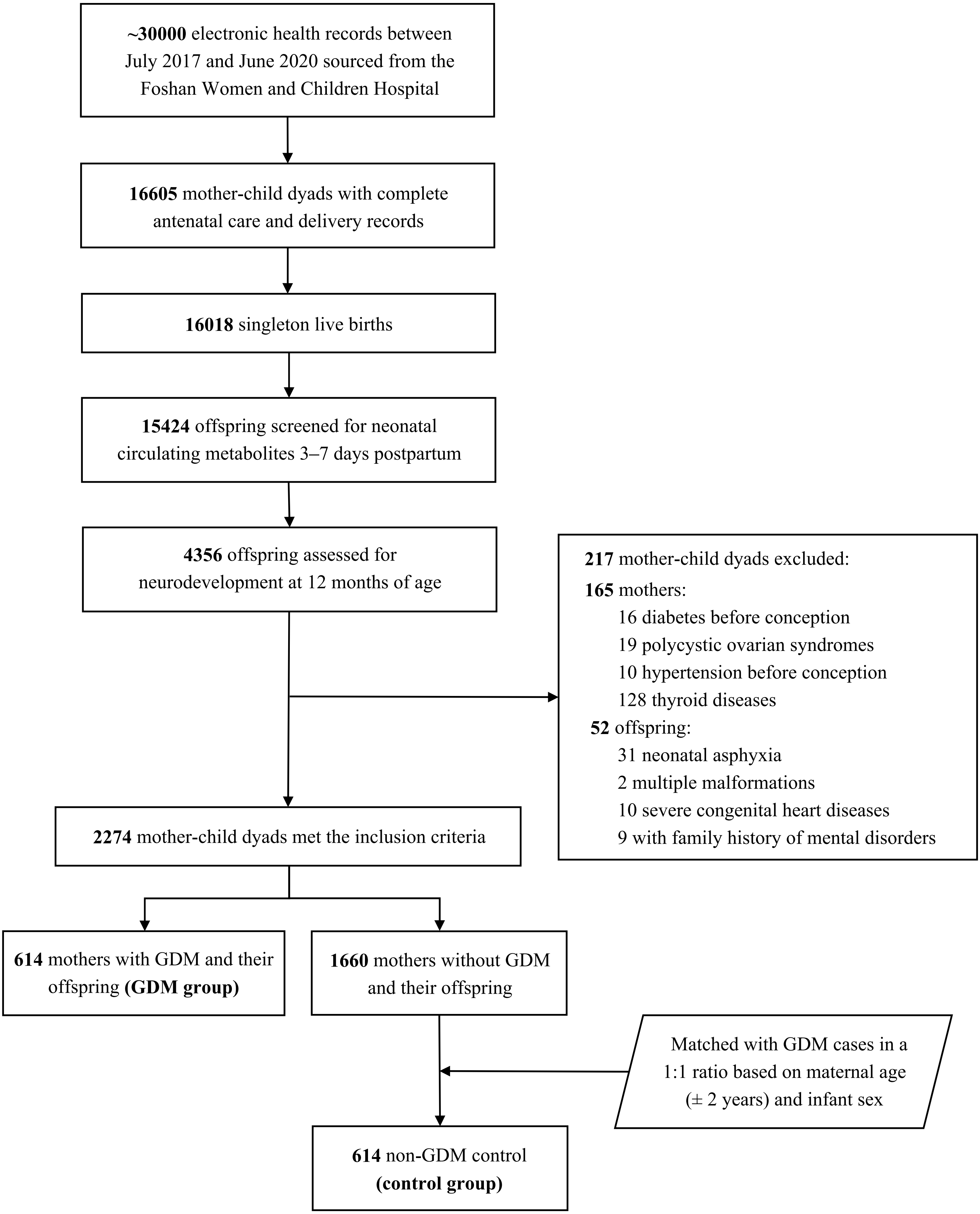

Figure S1. Selection profile of the study population. GDM, gestational diabetes mellitus.

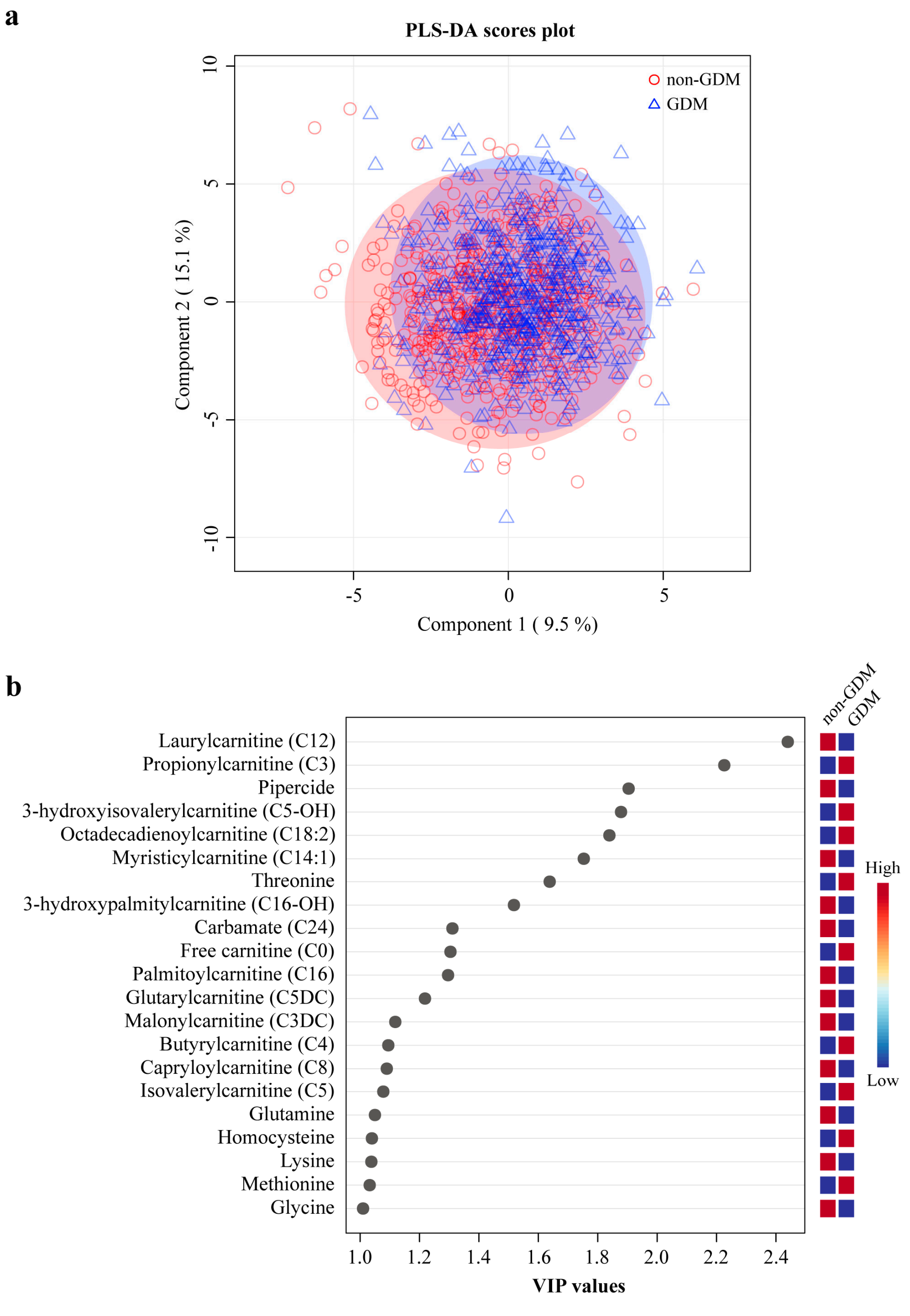

Figure S2. Comparison of neonatal circulating metabolite levels between GDM and non-GDM groups using the PLS-DA. **(a)** The 2-D PLS-DA scores plot reveals a partial separation across groups with a low risk of overfitting (p = 0.001 for 1000 permutation test). **(b)** A total of 21 neonatal circulating metabolites with VIP > 1.0 are identified. Metabolite data are log(e)-transformed to approximate a normal distribution and standardized as Z-scores before analysis. PLS-DA, partial least squares discriminant analysis; GDM, gestational diabetes mellitus; VIP, variance importance in projection.

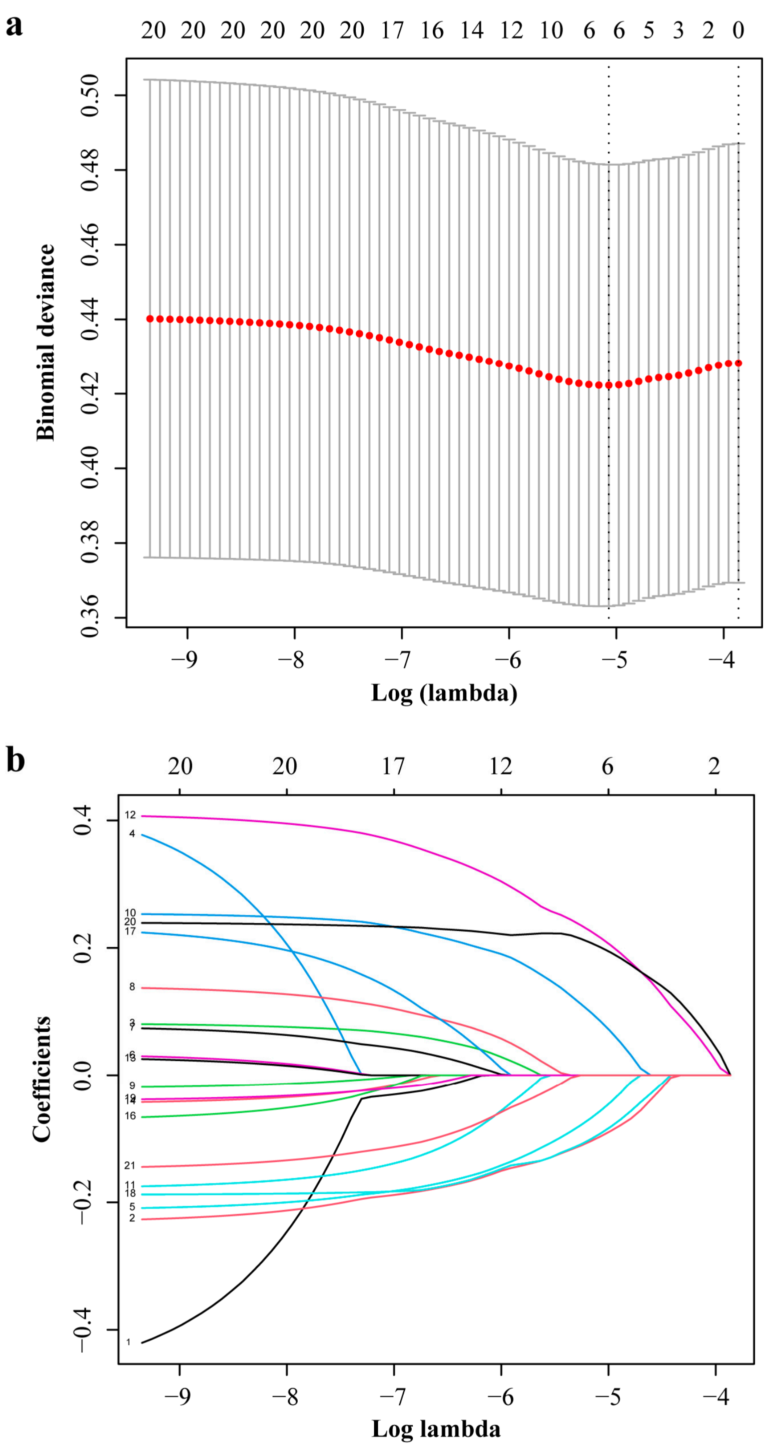

Figure S3. Selection of GDM-associated neonatal circulating metabolites for predicting offspring neurodevelopmental disorders at 1 year using the LASSO regression analysis with tenfold cross-validation. **(a)** Tuning parameter (lambda) selection of deviance in the LASSO regression based on the minimum criteria (left dotted line) and the 1-SE criteria (right dotted line). **(b)** A coefficient profile plot was created against the log (lambda) sequence. In our study, selection of metabolites was based on the minimum criteria (left dotted line), of which six metabolites with non-zero regression coefficients were selected. LASSO, least absolute shrinkage and selection operator; SE, standard error.

Variables	Odds ratio (95% confidence interval)	*p*-Value
GDM (GDM vs. non-GDM)	2.349 (1.388, 4.117)	0.002
Parity	0.487 (0.275, 0.831)	0.010
Infant sex (Femal vs. male)	0.644 (0.400, 0.997)	0.058
Birth weight	0.999 (0.998, 1.000)	0.010
Feeding patterns (Breastfeeding vs. non-breastfeeding)	0.195 (0.011, 0.914)	0.109

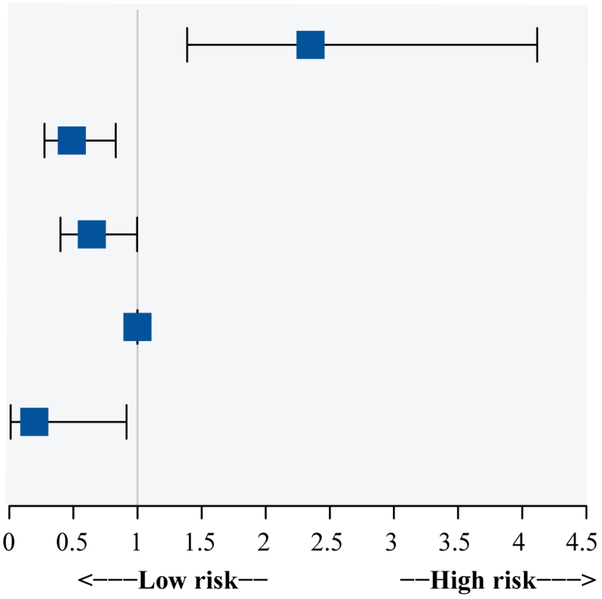

Figure S4. Selection of traditional risk factors for predicting offspring neurodevelopmental disorders at 1 year using the bidirectional stepwise logistic regression analysis. As a result, five traditional risk factors were retained in the stepwise regression model. GDM, gestational diabetes mellitus.

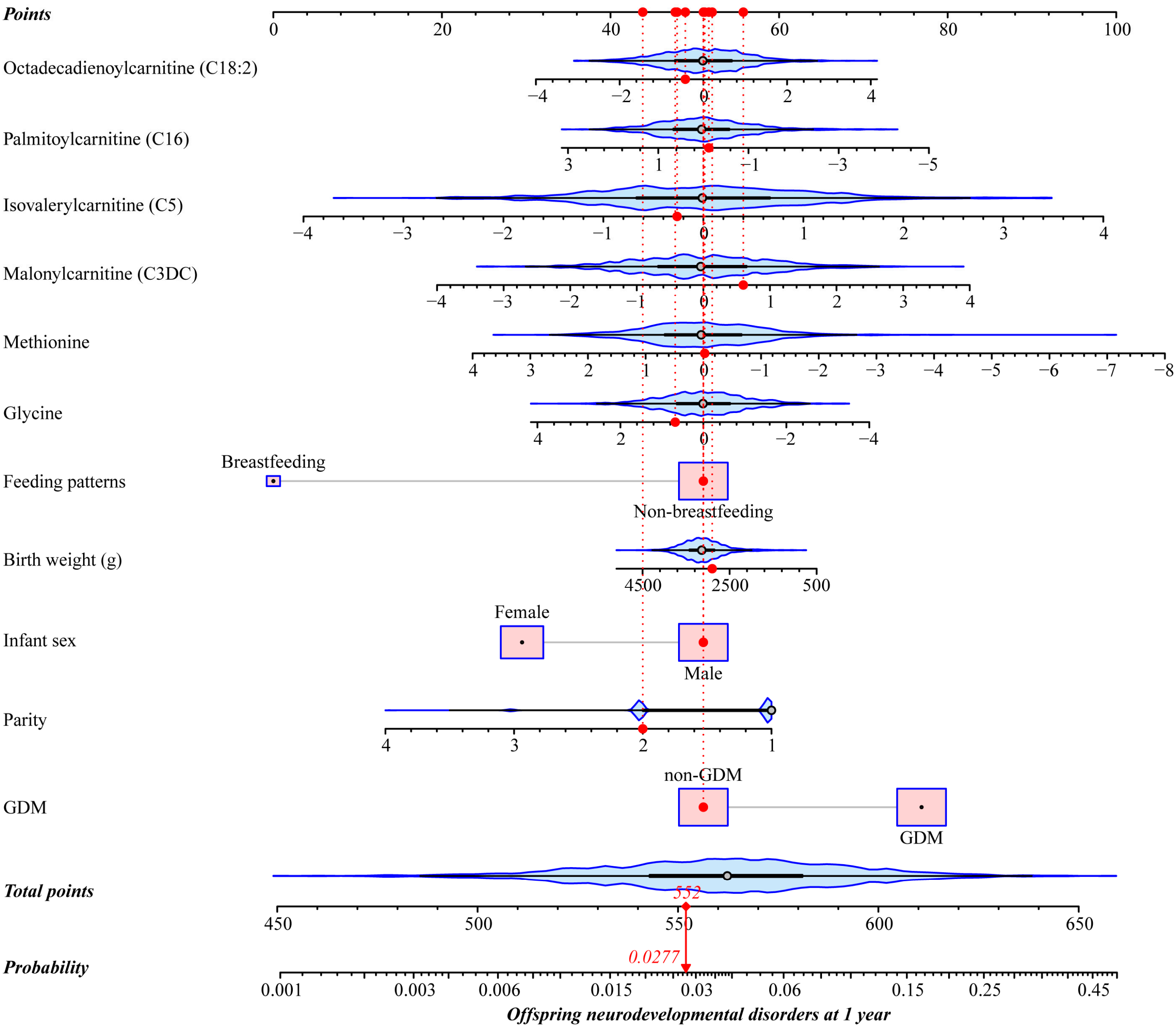

Figure S5. Nomogram depicting the combined model for predicting offspring neurodevelopmental disorders at 1 year. Metabolite data are log(e)-transformed to approximate a normal distribution and standardized as Z-scores before analysis. To use the nomogram, locate the observed value of each predictor variable for each mother-child dyad on the corresponding axis, draw a vertical line upward to the 'Points' axis to determine the point of each predictor variable, sum the points for all predictor variables and locate on the 'Total points' axis, and finally draw a vertical line down to the 'Probability' axis to find the probability of offspring neurodevelopmental disorders at 1 year. Take an example for the nomogram usage (a mother-child dyad is randomly selected from the study population): the mother is non-GDM, infant sex is male, parity is 2, birth weight is 2900 g, feeding pattern is non-breastfeeding, Z-score of log(e)-transformed neonatal glycine level is 0.678, Z-score of log(e)-transformed

neonatal methionine level is –0.024, Z-score of log(e)-transformed neonatal C3DC level is 0.600, Z-score of log(e)-transformed neonatal C5 level is –0.263, Z-score of log(e)-transformed neonatal C16 level is –0.121, and Z-score of log(e)-transformed neonatal C18:2 level is –0.431. Finally, a total point of 552 is obtained, and the corresponding probability for offspring neurodevelopmental disorders at 1 year is 2.77%. GDM, gestational diabetes mellitus.
